# The economic burden of disease of epithelial ovarian cancer in Spain: the OvarCost study

**DOI:** 10.1007/s10198-018-0986-y

**Published:** 2018-06-19

**Authors:** Laura Delgado-Ortega, Almudena González-Domínguez, Josep María Borrás, Juan Oliva-Moreno, Eva González-Haba, Salomón Menjón, Pedro Pérez, David Vicente, Luis Cordero, Margarita Jiménez, Susana Simón, Álvaro Hidalgo-Vega, Carlota Moya-Alarcón

**Affiliations:** 1 AstraZeneca Farmacéutica Spain, Serrano Galvache, 56, Building Álamo, Madrid Spain; 2 Weber, Majadahonda, Madrid Spain; 3 Clinical Sciences Department, University of Barcelona, L’Hospital de Llobregat, Barcelona Spain; 4 Universidad de Castilla-La Mancha – Campus de Toledo, Toledo, Spain; 5 Hospital General Universitario Gregorio Marañón, Madrid, Spain; 6 Hospital Universitario Virgen de las Nieves, Granada, Spain; 7 Hospital Clínico Universitario, Madrid, Spain; 8 Hospital Universitario Virgen Macarena, Sevilla, Spain

**Keywords:** Epithelial ovarian cancer, Economic burden of disease, Healthcare resource utilization, Spain

## Abstract

**Objective:**

To assess the economic burden of epithelial ovarian cancer (EOC) in incident patients and the burden by disease stage in Spain.

**Methods:**

We developed a Markov model from a social perspective simulating the natural history of EOC and its four stages, with a 10-year time horizon, 3-week cycles, 3% discount rate, and 2016 euros. Healthcare resource utilization and costs were estimated by disease stage. Direct healthcare costs (DHC) included early screening, genetic counselling, medical visits, diagnostic tests, surgery, chemotherapy, hospitalizations, emergency services, and palliative care. Direct non-healthcare costs (DNHC) included formal and informal care. Indirect costs (IC) included labour productivity losses due to temporary and permanent leaves, and premature death. Epidemiology data and resource use were taken from the literature and validated for Spain by the OvarCost group using a Delphi method.

**Results:**

The total burden of EOC over 10 years was 3102 mill euros: 15.1% in stage I, 3.9% in stage II, 41.0% in stage III, and 40.2% in stage IV. Annual average cost/patient was €24,111 and it was €8,641; €14,184; €33,858, and €42,547 in stages I–IV, respectively. Of total costs, 71.2% were due to DHC, 24.7% to DNHC, and 4.1% to IC.

**Conclusions:**

EOC imposes a significant economic burden on the national healthcare system and society in Spain. Investment in better early diagnosis techniques might increase survival and patients’ quality of life. This would likely reduce costs derived from late stages, consequently leading to a substantial reduction of the economic burden associated with EOC.

**Electronic supplementary material:**

The online version of this article (10.1007/s10198-018-0986-y) contains supplementary material, which is available to authorized users.

## Introduction

Ovarian cancer (OC) is a rare disease but with a high mortality rate in women [1]. In 2012, the estimated number of new cases in Europe was 65,538 and accounted for a total of 42,716 deaths [2]. That year, the incidence and mortality of OC in Spain were estimated between 13.7 and 7.9 per 100,000 population, being the fifth most frequent cancer type in women and the sixth leading cause of mortality [1].

It is a heterogeneous disease and has many histological subtypes; however, the majority of cases (~90%) are of epithelial origin (EOC) [3]. The cause of OC is unknown, but many associated risk factors have been identified. It is predominantly a disease diagnosed in postmenopausal women with the majority of cases (> 80%) being diagnosed in women over 50 years [3]. A woman’s reproductive history appears to contribute significantly to her risk of ovarian cancer, although the family history also plays an important role. Approximately, 11–15% of OC are associated with inherited predisposition, mainly related to germline mutations in *BRCA1*/*2* genes [4]. Age also constitutes a risk factor in those OC patients with *BRCA1*/*2* mutations, with the mean age of onset being significantly earlier in those with a *BRCA1* mutation (45 years) compared with over 60 years of age for those with a *BRCA2* mutation [5].

Due to the non-specific symptomatology of the onset and despite continuous advances in hereditary OC identification to prevent it, most patients (75%) [6, 7] are diagnosed with an advanced stage of disease according to the International Federation of Gynaecology and Obstetrics (FIGO) classification [8]. Staging is related to survival and is the most important factor to assess the prognosis of the patient. According to the FIGO Annual Report, women diagnosed with EOC between 1999 and 2001, had a 5-year survival mean rate of 86.4% among those diagnosed at stage I, 69.9% for those at stage II, 34.3% at stage III, and 18.6% for those diagnosed at stage IV [9].

EOC has a major impact on patients’ quality of life and implies an important economic burden for healthcare services, patients, and society in general, for several reasons. These patients are treated with a large and growing amount of healthcare resources such as hospitalizations, medical appointments, and chemotherapy treatments administrated in day hospital units, since they are diagnosed [10, 11]. Administration is usually expensive, not only because of medical resource consumption, but also because it requires time expenditure from experienced nurses on day hospital units [9, 12, 13]. Additionally, the own aetiology of the disease entails a high risk of hospitalization [14]. Also, women diagnosed with EOC are usually of working age, so labour productivity losses due to premature mortality and to permanent and temporary leaves are, therefore, deemed considerable [15, 16]. In addition, patients in their last stages are likely to require home care, usually provided by family members [17]; professional care and support activities provided by informal caregivers have a relevant opportunity cost, which from a societal perspective should be accounted for.

Despite the considerable costs described above, the economic burden of EOC from a societal perspective had been scarcely analysed in the international literature and, specifically, in Spain. Measuring this burden may be relevant for healthcare decision makers, as it provides useful information to assess the real magnitude of the benefits derived from the possible intervention programs and health strategies targeting the disease. Moreover, it offers a baseline for prevention policy planning, and health resources and social care allocation.

Therefore, the main objective of this study was to assess the economic burden of EOC in incident patients in Spain, as well as the burden by disease stage. It provides essential evidence about resource cost, their evolution over time, and the efficiency of new treatments at each disease stage for economic evaluations. The secondary objective was to raise awareness about the importance of this cancer among society and healthcare authorities.

## Methods

A Markov model was considered as the most appropriate method to simulate the progression of EOC, regarding the modelling approaches adopted in the previous economic studies and the nature of the disease [18, 19]. A societal perspective was adopted and only incident cases of EOC in Spain were included.

Epidemiology data, survival rates, healthcare resources used, personal care (formal and informal), and productivity losses to populate the model were obtained from a literature review, including international and national references. International data were used whenever local data were not available. Databases consulted were Medline/Pubmed, Embase, Medes, American Economic Association’s Electronic Bibliography (EconLit), and other official databases.

All extracted data were afterwards contrasted and validated through a multidisciplinary expert group using the Delphi methodology. This included one individual online survey and two in-person meetings to reach final consensus. The OvarCost Expert Panel was composed of a gynaecologic oncologist, a clinical oncologist, a genetic counselling specialist, an oncology hospital pharmacist, a health economics specialist, and an epidemiologist involved in cancer management at regional and national level.

**Fig. 1:**
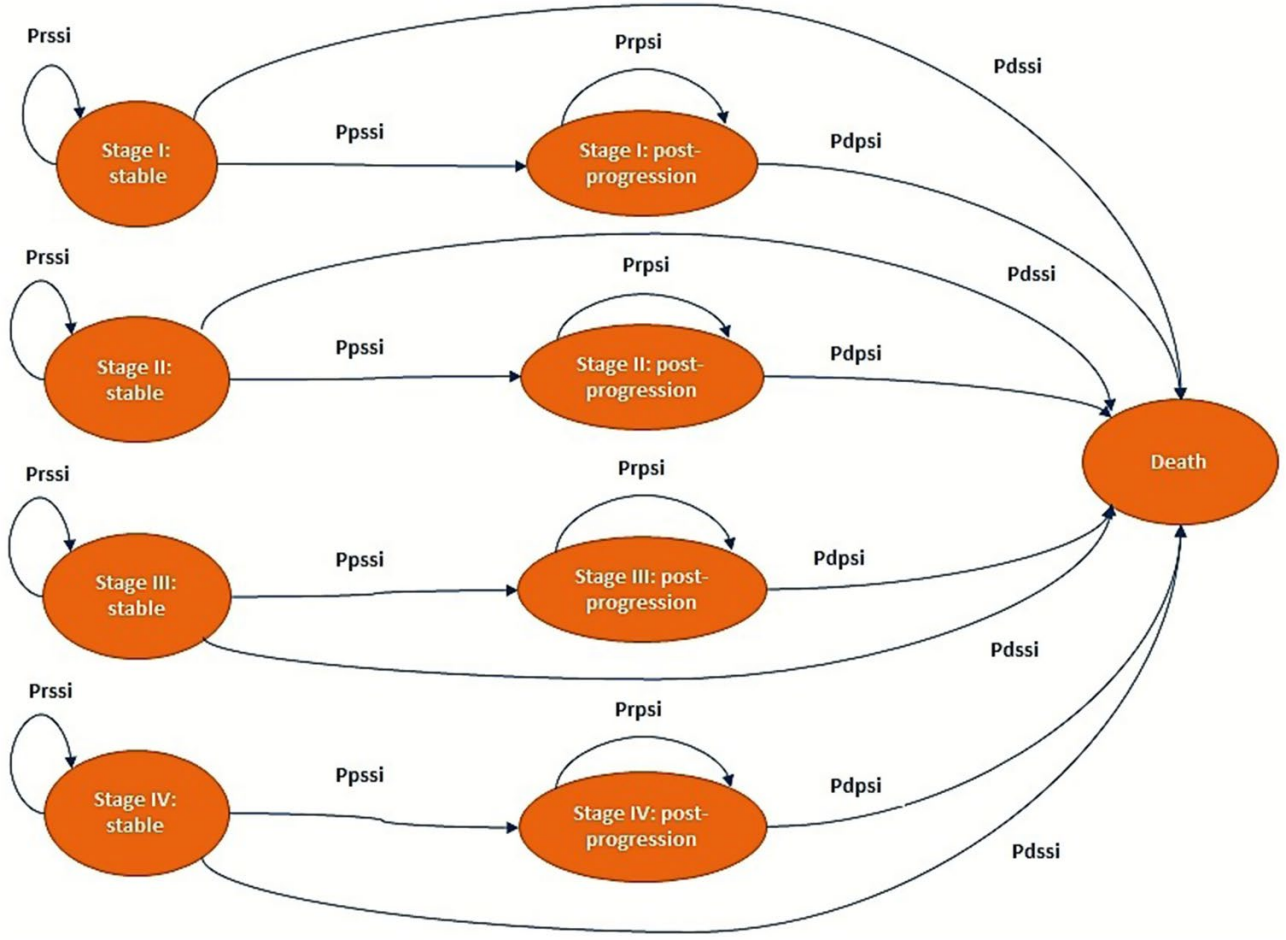
Markov model structure *i* disease stages I, II, III, or IV. *Prssi* probability of remaining in “stable” state (Stage i), *Pdssi* probability of dying in “stable” state (Stage i), *Ppssi* probability to progress from “stable” state (Stage i), *Prpsi* probability of remaining in “post-progression” state (Stage i), and *Pdpsi* probability of dying in “post-progression” state (Stage i)

The Markov model was developed with three possible health states: *stable, post-progression*, and *death* (Fig. 1). The time horizon of the model was 10 years, which is enough considering the survival rate of the disease and all the cost and clinical consequences for all four stages. The cycle length used was 3 weeks (21 days), which is the length of a chemotherapy cycle. Patients entered the model after they were diagnosed with EOC in the *stable* state. A cycle after, they can either remain *stable*, become worse, and move to a *post-progression* state, or die. Those who progress remain in a *post-progression* state until they die. Mortality risk in patients may change depending on their health state (*stable* or *post-progression*) and their disease stages (I, II, III, and IV) (Fig. 1). It is considered that patients are allocated to a specific disease stage at the initial diagnosis, and it does not change throughout their disease.

### Population

Incident patients were estimated from years 2017 to 2026, using a linear model between 2015, 2020, and 2025 as per GLOBOCAN predictions [1]. Accordingly, 3497 women diagnosed with ovarian cancer were estimated for the first year, and as per epidemiology data, the majority of cases (90%) are of epithelial origin [3]. Those were distributed by the four disease stages [20], as shown in Table 1. New cases diagnosed in the following years until 2026 were added each year assuming no changes in the distribution of disease stages over time. Incident patients’ distribution by stage comes from population-based cancer registries [20].

**Table 1:** Epidemiology, patient characteristics, and treatment of EOC by disease stage

	Stage I	Stage II	Stage III	Stage IV
*Epidemiology of EOC*				
EOC incidence (*n*, (% of total))	1155 (37%)	195 (6%)	1116 (35%)	681 (22%)
Median progression-free survival (years)*	18.33	6.25	2.00	1.60
Median overall survival (years)*	19.50	7.50	3.20	1.90
*Patient characteristics*				
Mean age at diagnosis (years)	57.4	62.4	64.9	68.1
Mean weight (kg)	65	67	65	66
Mean height (cm)	159	160	159	160
*Hospitalizations and emergencies every* 6 *months*				
Number of hospitalizations	1.2	1.2	2.1	2.1
Patients hospitalized (%)	15.4	15.4	48.2	48.2
Number of emergencies	1.5	1.5	1.7	1.7
Patients in emergency services (%)	23.1	23.1	22.2	22.2
*Treatment*				
None	0%	0%	3.10%	8.80%
Surgery	66.70%	19.80%	11.30%	8.80%
Neoadjuvant chemotherapy + surgery	0%	0%	14.40%	24.20%
Surgery + adjuvant chemotherapy	33.30%	80.20%	71.10%	58.20%
*Type of surgery*				
Laparotomy	100%	100%	100%	100%
Omentectomy	0%	6.38%	100%	100%
Abdominal total hysterectomy	100%	100%	100%	100%
Bilateral salpingo-oophorectomy	0%	100%	100%	100%
Lymphadenectomy	0%	0%	75%	100%

*Own elaboration based on Heintz et al. [9]

*EOC,* epithelial ovarian cancer

### Transition probabilities

Transition probabilities depend on the disease stage assigned at diagnosis and the health state as patients enter the model. Death probability in the *stable* state (Pdss) was the mortality rate in the general Spanish female population [21] (Pnd, natural death probability). This mortality rate was estimated taking into account age at diagnosis and at each stage of the disease [15], as stated in Table 1, and its evolution over time; finally, it was transformed to probabilities as 1 − exp^(rate at age)^.

**Fig. 2:**
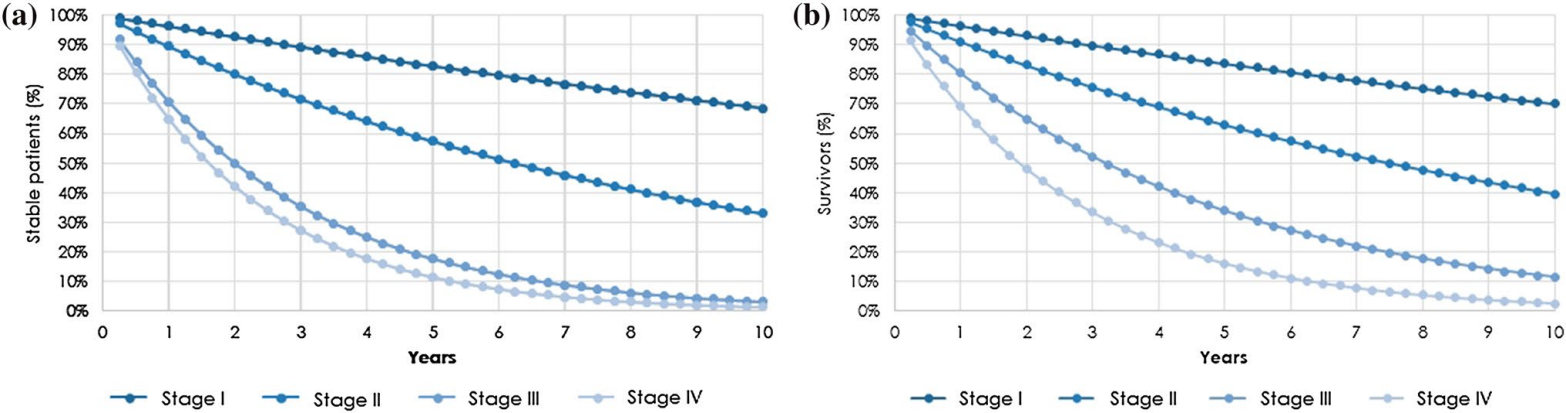
Survival curves by disease stage. Progression free survival (**a**) and overall survival (**b**)

Transition probabilities from *stable* state to *post-progression* state (Ppss) (Eq. 1) were assessed by progression-free survival (PFS) curves for each disease stage, which were built based on an exponential distribution of median PFS (Table 1; Fig. 2a) [9].

Equation 1. Probability of transitioning from *stable* to *post-progression* state:

1 $${\text{Ppss}}=1 - \exp ( - \lambda t);\,{\text{with}}\,\,\,\,\lambda = - \frac{{\ln \left( {0.5} \right)}}{{{\text{m}}{{\text{e}}_{{\text{PFS}}}}}}.$$

Transition probabilities of patients who remain *stable* (Prss) were calculated as the inverse of the probability of dying plus the transition probability of progressing from *stable* to *post-progression* state. Transition probabilities from *post-progression* to *death* (Pdps) were estimated as the difference between overall survival (OS) and PFS, plus the probability of natural death (Eq. 2) (Fig. 2b). OS curves were built using an exponential distribution, taking into account the median OS at each stage published by Heintz et al. [9] (Table 1; Fig. 2b).

Equation 2. Probability of dying for patients at *post-progression* state (Pdps):

2 $${\text{Pdps}}=1 - \exp \left( { - {\lambda ^\prime }t} \right)+{\text{Pnd}};\,{\text{with}}\,\,\,{\lambda ^\prime }= - \frac{{\ln \left( {0.5} \right)}}{{{\text{m}}{{\text{e}}_{{\text{OS}}}}~ - ~{\text{m}}{{\text{e}}_{{\text{PFS}}}}}}.$$

Transition probabilities of the patients who remain at the *post-progression* state (Prps) were estimated as the inverse of the probability of dying for patients at *post-progression* state.

### Costs and resource use

Costs were expressed in 2016 Euros (Tables 1, 2 in Online Resource). Unit healthcare costs were the median value of the unit costs for each Autonomous Community in Spain [20–37]. Since the costs come from different years, they were updated to € 2016 using the corresponding inflation rate: a medicine consumer price index (CPI) of 0.77% for direct healthcare costs (DHC) [40] (except for pharmaceutical costs and tariffs from Autonomous Communities already actualised) and a general CPI increase of 1.97% for direct non-healthcare costs (DNHC) [40]. In the Markov model, the costs were discounted at an annual rate of 3%, according to Spanish health technology assessment recommendations [41]. An annual growth rate of 1% was considered for labour productivity loss [42]. The average annual cost per patient was assessed dividing the total cost by the number of patients (those who were alive at the beginning of the year plus the incident patients in that year). Model costs were categorised as DHC, DNHC, and indirect costs (IC).

DHC included diagnosis and follow-up tests, treatments, and palliative care. Testing required at diagnosis according to the Spanish Society of Gynaecology and Obstetrics [12] include ovarian biopsy, biochemical analysis and vaginal ultrasonography, among others. Regarding selection criteria to identify *BRCA* mutation, it was agreed by the panel group that 20% of patients with EOC are referred to *BRCA1*/*2* genetic test and genetic counselling, accounting two of these visits, before and after the test [43]. Of these patients, 5% are identified with the genetic mutation and an average of five family members are derived to genetic counselling [44], a transvaginal ultrasound and a blood test every 6 months to detect the tumour marker CA125 [43]. The patient’s follow-up depends on their disease state. The frequency for follow-up testing may be lower in stable patients, depending on the period of time that they remain at this health estate [12]. The percentage of patients hospitalized and those who attend to the emergency department due to EOC were also considered by disease stage (Table 1) [14]. Treatment management depends on many factors, such as the spread of the tumour and the patient’s clinical situation, being surgery or/and chemotherapy the standard of care [3, 9]. Treatment usually starts with the surgical excision of the tumour mass. Nevertheless, this procedure is not always possible and an interval debulking surgery is performed. This intervention is a surgical excision that takes place after patients have taken neoadjuvant chemotherapy [12]. Most patients receive adjuvant chemotherapy. However, patients in stages Ia and Ib do not need chemotherapy after surgery and only remain under clinical observation [12]. The type of surgery depends on the size and the spread of the tumour, and on whether or not the woman is planning to get pregnant in the future [12, 13].

**Table 2:** Results of the model: direct healthcare costs, direct non-healthcare costs, and indirect costs by stage

	Stage I		Stage II		Stage III		Stage IV	
	Total cost, € (%)	Average annual cost/patient (€)	Total cost, € (%)	Average annual cost/patient (€)	Total cost, € (%)	Average annual cost/patient (€)	Total cost, € (%)	Average annual cost/patient (€)
Direct healthcare costs (DHC)								
Opportunistic screening test (patients)	3,088,335 (0.66)	88.90	519,790 (0.45)	92.46	2,984,717 (0.23)	103.10	2,569,116 (0.21)	119.03
Genetic counselling (relatives)	1,477,440 (0.32)	26.63	248,570 (0.21)	28.78	1,427,697 (0.11)	35.33	1,105,642 (0.09)	35.09
Diagnosis	6,845,283 (1.46)	197.04	1,152,114 (0.99)	204.94	15,650,937 (1.23)	540.63	13,471,654 (1.08)	624.17
Follow-up visits and test	12,550,184 (2.68)	230.70	2,448,326 (2.10)	288.28	16,131,636 (1.27)	414.67	7,449,071 (0.60)	260.89
Surgery	33,386,327 (7.12)	794.29	9,138,573 (7.84)	1,550.41	87,624,527 (6.89)	2,879.72	73,843,438 (5.93)	3,211.65
Chemotherapy	73,429,456 (15.66)	1,399.93	28,018,052 (24.02)	3,517.86	223,313,473 (17.57)	6,274.45	725,107,595 (58.21)	23,527.41
Hospitalizations	104,089,546 (22.20)	1,915.55	15,453,906 (13.25)	1,879.60	377,473,449 (29.69)	10,199.17	315.441.267 (25.32)	10,197.08
Emergency services	6,240,086 (1.33)	114.84	926,449 (0.79)	112.68	4,249,286 (0.33)	114.81	3,550,979 (0.29)	114.79
Palliative care	4,793,722 (1.02)	76.47	1,912,383 (1.64)	203.48	21,116,270 (1.66)	515.37	10,077,388 (0.81)	527.30
Total DHC	245,900,380 (52.45)	4,844.35	59,818,164 (51.29)	7,878.49	749,971,992 (59.00)	21,077.26	1,152,616,149 (92.54)	38,617.41
Direct non-healthcare costs (DNHC)								
Public formal care	126,432 (0.03%)	1.99	50,370 (0.04%)	5.30	553,693 (0.04%)	13.39	259,010 (0.02%)	13.33
Private formal care	1,235,043 (0.26%)	19.45	492,033 (0.42%)	51.74	5,408,711 (0.43%)	130.82	2,530,115 (0.20%)	130.23
Informal care	112,708,919 (24.04%)	1,784.14	47,855,777 (41.03%)	5,049.87	505,313,452 (39.75%)	12,293.17	90,186,328 (7.24%)	3,785.87
Total DNHC	114,070,394 (24.33%)	1,805.58	48,398,180 (41.50%)	5,106.91	511,275,856 (40.22%)	12,437.38	92,975,453 (7.46%)	3,929.43
Indirect costs (IC)								
Temporary disability	37,936,056 (8.09%)	973.23	4,136,727 (3.55%)	668.13	9,975,732 (0.78%)	342.54	0 (0%)	0
Permanent disability	6,129,363 (1.31%)	102.23	773,669 (0.66%)	94.37	0 (0.00%)	0.00	0 (0%)	0
Premature mortality	64,769,698 (13.82%)	915.14	3,496,382 (3.00%)	435.63	10,774 (0%)	0.38	0 (0%)	0
Total IC	108,835,116 (23.22%)	1,990.61	8,406,778 (7.21%)	1,198.13	9,986,506 (0.79%)	342.92	0 (0.00%)	0
Total	468,805,889 (100%)	8,640.53	116,623,122 (100%)	14,183.54	1,271,234,354 (100%)	33,857.56	1,245,591,602 (100%)	42,546.84

Clinical experts panel classified chemotherapy as: (1) neoadjuvant: patients who receive 3 cycles of paclitaxel in combination with carboplatin before surgery and complete their treatment with other 3 cycles of chemotherapy [12]; (2) adjuvant: chemotherapy administered after surgery in the *stable* state (stages I, II, and III); (3) post-progression: chemotherapy administered at the *post-progression* state (stages I and II); and (4) advanced: chemotherapy administered at the *post-progression* states at stage III and at both states at stage IV (Table 1).

The recommended drugs used are based on the EOC treatment recommended by SEGO guidelines [12]: paclitaxel, carboplatin, doxorubicin, bevacizumab, cisplatin, gemcitabine, topotecan, trabectedin, and docetaxel. Its usage was accounted based on its market share [45]. Doses were calculated according to the usual clinical practice and product labels [46–54] (Table 4 in Online Resource). Dose of carboplatin [47] was determined using the Calvert formula [55]. Du Bois et Du Bois formula was used to calculate the body surface area when necessary [56] (Table 3 in Online Resource). Patients’ height and weight were consulted in Spanish National Health Survey according to the mean age at diagnosis of each stage disease [15, 57] (Table 1). Drug costs were calculated using the list price (LP) [58], including Royal Decree Law 8/2010 deduction rate, when necessary, and a 4% of the value-added tax (VAT) entitled for Spain [58–61]. For intravenous drugs, the model also considered non-vials optimization and the cost of administration for each drug (€0.32 per minute [62]): time of administration required for each one [46–53] in the day hospital plus the cost of the 30-min preparation (Table 3 in Online Resource).

Palliative care is given to the patients in their last 48 days of life [63]. Up to 93.3% of the patients receive follow-up care at outpatient hospitals, while the remaining (6.7%) are assisted by palliative home care team [64]. Patients need a mean of 9.5 home visits of palliative-care services, while those who receive follow-up at the outpatient hospital are seen by a nurse [63]. In both cases, patients also pay four visits, on average, to the primary-care doctor [63] and they spent their terminal phase of their illness at home (59.6%) or at the hospital (40.4%) [65]. The last 3 days of this terminal phase, patients stay at home [66], and they are visited twice a day by a nurse [67]. Of these, 14% receive sedation [66]. The costs of visits [22–39] and drugs used in the palliative-care phase were also considered [58–61, 68–71] (Tables 1, 4 in Online Resource).

DNHC considered were formal care costs (i.e., professional care financed by private or public funds) and informal care costs given at home (non-remunerated care from relatives or friends). Based on the literature, it was assumed that 17.4% of the patients received private care, 9.5% public care [63], and 93.4% received informal care [72] throughout their last 48 days of life [63]. On average, it was considered that public caregivers spent 1.5 h providing care [63], private caregivers 8 h [63], and informal caregivers 10.3 h [17]. According to the proxy good method [73], hourly wage for formal and informal caregivers was equally valued, €13.56 [74].

Lost labour productivity due to temporary or permanent leave and premature death were included as indirect costs (IC), using the human-capital method [75–78]. At some point in the progress of the disease, patients with EOC become unable to develop their labour activities [79, 80]. Overall, 30% of *stable* patients lose 60 days due to temporary leave, while the remaining 70% lose over 70 days [45]. It was assumed that at advanced stages, patients are on sick leave, since progression starts until age of retirement (65 years). In case the patient’s death occurs before 65 years, sick leave period is assumed to last between the beginnings of the disease progression until patient dies. Patients on sick leave for more than 1 year were considered to be in permanent leave [81]. Labour productivity loss caused by premature death included lost productivity of patients who die before 65 years of age [21, 79, 82, 83]. The percentage of women employed and their respective salaries are used to estimate labour productivity losses (Table 5 in Online Resource).

### Sensitivity analysis

Deterministic and univariate sensitivity analyses, including ten different scenarios, were conducted to examine the model’s robustness. According to the OvarCost Expert Panel, different scenarios were built based on the possible variation of the most sensitive parameters: percentage of patients who receive genetic counselling (from 35 to 70%), patients weight (± 10%), growth productivity discount rate on (from 0 to 2%), manufacturer’s drug price (− 10%), discount drug rate (from 0 to 6%), tests and medical visits cost (maximum and minimum prices in the Autonomous Communities), age at time of diagnosis (± 10%), bevacizumab dose recommendation (7.5 mg/kg), caregiver’s salary/informal care assessment per hour (€7.5) [84], and informal care hours received (± 30%).

## Results

**Fig. 3:**
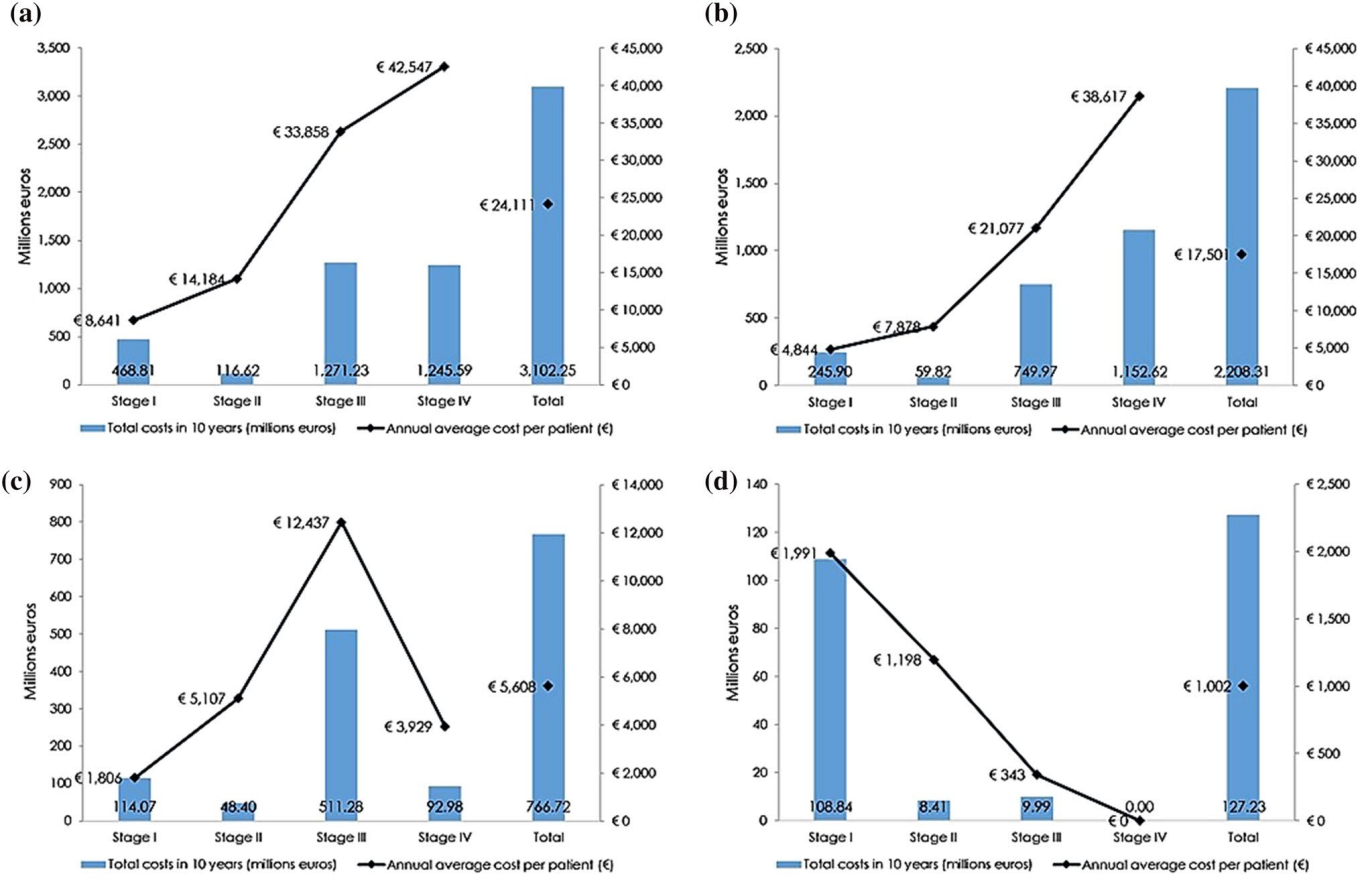
Cost distribution results by disease stage. Total costs (**a**), direct healthcare costs (**b**), direct non-healthcare costs (**c**) and indirect costs (**d**)

The total economic burden of EOC in Spain was estimated in 3102 million euros (mill€) in 10 years. Stages I and II represented around 19% of the total cost of the disease each year, while stages III and IV accounted for 41 and 40%, respectively (Table 2). The average annual cost per patient was €24,111, being the greatest cost the corresponding to patients in stage disease IV (€42,547). By cost type, most of the economic burden of EOC was due to DHC (71.2%) being the DNHC 24.7% and IC 4.1% of the total, since most patients are diagnosed over 40 years of age. However, IC are more relevant at early stages (23.2% at stage I and 7.2% at stage II) (Fig. 3a; Table 2).

DHC were estimated in 2208 mill€ in 10 years. The average annual cost per patient was €17,501. The most important cost categories were advanced chemotherapy (909.9 mill€), hospitalizations (812.5 mill€), and surgery (204.0 mill€). Together, these represented around 87.2% of DHC. The average annual cost per patient was €6657.6 for advanced chemotherapy; €6304.1 for hospitalizations, and €2024.6 for surgeries (Table 2). Average annual costs per patient increased as the cancer spread, with DHC at stage IV being around eight times higher than DHC at stage I (Fig. 3b).

DNHC represented 766.7 mill€ in 10 years. The average annual cost per patient was €5608. Patients at stages II, III, and IV were substantially assisted by informal carers. Informal care implied 98.6% of DNHC at any stage (Table 2) and reached €12,437.4 per patient per year at stage III (Fig. 3c).

Lost labour productivity was estimated in 127 mill€ in 10 years, of which temporary leave accounted for 40.9%; permanent leave for 5.4%, and premature death for 53.7%. Most losses occurred at early disease stage, with 85.5% in stage I (Fig. 3d). Labour productivity losses amounted to €1002.1 per patient every year. IC annual per patient was €1990.6 at stage I and €1198.1 at stage II. However, a lower productivity cost was observed for patients at stages III and IV (€342.9 at stage III and € 0 at stage IV) (Table 2).

### Sensitivity analysis

The sensitivity analysis was based on the percentage of patients who receive genetic counselling, patients weight, discount rate on growth productivity, and manufacturer price had almost no impact on the global burden of the disease (changed the average annual cost per patient in between − 2.9 and 2.6%).

**Fig. 4:**
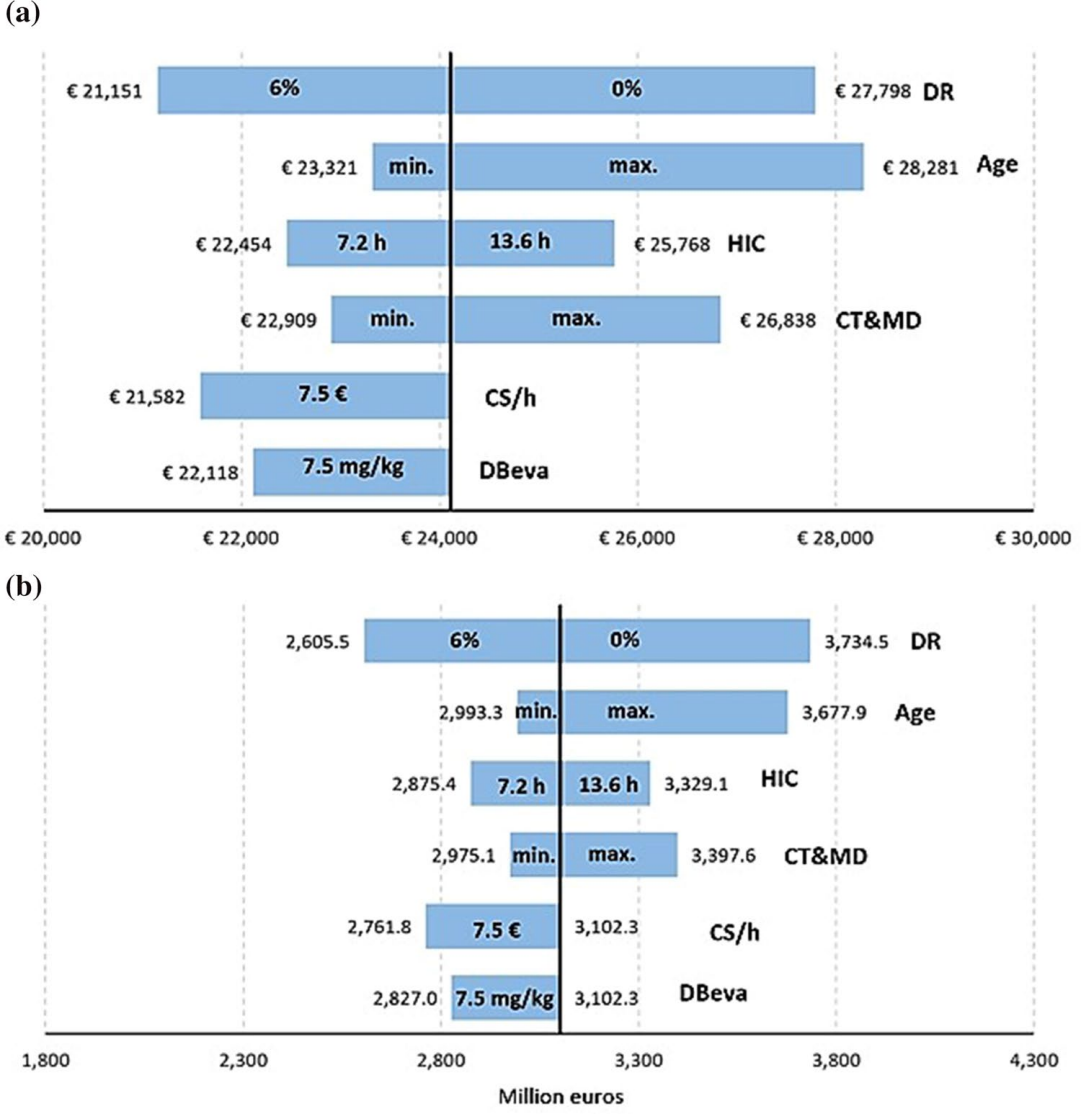
Total cost tornado diagram (a) and the average annual cost per patient of epithelial ovarian cancer (**b**). *DBeva* dose of bevacizumab, *HIC* hours of informal care, *CS/h* caregiver salary per hour, *CT and MD* cost of tests and medical visits, *DR* discount rate on growth productivity

Figure 4 shows sensitivity analysis results which substantially modified the global study results. The average annual cost per patient (€24,111) fluctuates between €21,151 and €28,281, which influences the global burden between 2605 and 3734 mill€ in 10 years, being in the base case analysis 3102 mill€.

## Discussion

To our knowledge, this is the first study that is close to estimating the economic impact of EOC in Spain considering DHC as well as DNHC and IC involved in patient care. Our estimates indicate that the global average cost per patient with EOC may amount to €24,111 every year in Spain, with significant differences by disease stages, from €8,641 at stage I to €42,547 at stage IV. This result was expected as most of the patients are diagnosed at advanced stages of the disease and those are likely to need more healthcare resources and home care.

Research on the economic burden of OC is scarce in scientific literature. However, Kim et al assessed the annual DHC per patient in Hungary, Serbia, and Slovakia [18]. Their approach was based on different health states: surgical treatment; first-line, second-line, and third-line chemotherapy; and monitoring/follow-up and palliative care/death. However, we calculated the DHC per stage of disease, according to the FIGO classification [8]. This latter methodology allows estimating the resources that patients require, considering their stage at diagnosis. It also makes easier the comparison to future studies regarding the burden of OC in other countries.

The growing scientific literature on informal care costs suggests the relevance of this social resource in the case of many diseases and injuries [85, 86]. One of the strongest findings of our study is the estimation of informal care costs associated with EOC. To our knowledge, this is the first study that estimates the economic impact of caregiving among EOC patients in Spain. Our results show that costs of informal care represent 24.4% of the global burden of EOC, and are higher in stage III. Finally, we found that IC represent the smallest proportion of the global burden (4.1%), and they are significantly higher at early stage of the disease (stage I), when women are more likely to be of working age and less likely to die prematurely, than at later stages.

Our study is not without limitations. First, due to the lack of information about this cancer in Spain, the PFS curves from other countries were deemed similar enough to be used for our country. In addition, some data referring to all types of OC were used, since EOC accounts for 90% of the OC in general. Those data were considered representative and valid. Second, as neither national nor international references were found regarding average height and weight of patients with EOC by disease stage, our model included the average weight and height of women with any cancer by age at diagnosis in Spanish National Health Survey. However, the sensibility analysis showed that the weight of the patients had almost no impact on the global burden of EOC. Third, because of this lack of data availability about the patients in Spain, we adopted an incidence model approach. However, since this method does not include the patients previously diagnosed, and it may underestimate the burden of EOC. Fourth, our model considered that vials were used only once, although in Spanish practice, patients are usually gathered in day hospitals to optimise drug vials usage. Optimization of vials would decrease the global cost of treatment. Fifth, the percentage of patients who needed informal care was obtained from an observational study about patients with haematological neoplasia developed in Spain, whose situation may be different compared to those with EOC. Finally, our study does not quantify the substantial psychological load that caregivers may suffer from.

Despite its limitations, we believe that this study represents the most complete economic burden of EOC performed to date in Spain. Our results suggest that the disease’s economic impact on healthcare resources significantly increases with the stage at which the cancer is diagnosed. Investment in the development and evaluation of techniques for early diagnosis may imply higher survivals rates and a substantial reduction in the economic burden of EOC, due to possible cost savings at advanced disease stages. Besides, this study emphasizes the importance of informal care in the global burden of the disease, especially in advanced stages. In conclusion, our results highlight the importance of analysing the economic consequences of EOC from a societal perspective, providing an insight into the distribution of this cancer costs by stage, with the final aim of informing healthcare services planning appropriately.

## Electronic supplementary material

Below is the link to the electronic supplementary material.

Supplementary material 1 (DOCX 36 KB)
