# Supplementary material for: The economic burden of disease of epithelial ovarian cancer in Spain: the OvarCost study
Source: Eur J Health Econ. 2018 Jun 19;20(1):135–47. doi: 10.1007/s10198-018-0986-y (PMC6394604; doi:10.1007/s10198-018-0986-y)
Supplement: Supplementary file 1 — Supplementary material 1 (DOCX 36 KB) [file 10198_2018_986_MOESM1_ESM.docx]

Article title:

THE ECONOMIC BURDEN OF DISEASE OF EPITHELIAL OVARIAN CANCER IN SPAIN. THE OVARCOST STUDY

Journal name:

The European Journal of Health Economics

Author names:

Author name abbreviattion and order in citation:

Delgado-Ortega L, González-Domínguez A, Borras J, Oliva J, González-Haba E, Menjón S, Pérez P, Vicente D, Cordero L, Jiménez M, Simón S, Hidalgo A, Moya-Alarcón C

Full name of authors:

- Laura Delgado-Ortega
- Almudena González-Domínguez
- Josep María Borrás
- Juan Oliva-Moreno
- Eva González-Haba
- Salomón Menjón
- Pedro Pérez
- David Vicente
- Luis Cordero
- Margarita Jiménez
- Susana Simón
- Álvaro Hidalgo-Vega
- Carlota Moya-Alarcón

Affiliation and e-mail address of the corresponding author:

Name: Laura Delgado-Ortega; Affiliation: AstraZeneca Farmacéutica Spain S.A.; E-mail address: laura.delgado@astrazeneca.com

**Supplementary Material**

Table 1. Direct healthcare costs per unit.

|  | Costs (€, 2016) | References |
| --- | --- | --- |
| Screening tests and genetic counselling | | |
| CA-125 | 13.18 | [26, 27, 32, 33, 39] |
| Genetic counselling (1st visit) | 229.44 | [32, 34, 39] |
| Genetic counselling (subsequent visits) | 114.72 | [32, 34, 39] |
| Transvaginal ultrasound | 43.22 | [23, 31, 36–38] |
| BRCA 1/2 genetic test | 1,192.84 | [32] |
| Diagnosis and follow-up tests | | |
| Biopsy | 118.90 | [22, 24, 26–39] |
| Biochemical analysis | 24.94 | [24, 27, 28, 32, 33, 35, 38, 39] |
| CA 19-9 | 14.07 | [26, 27, 32, 33, 38, 39] |
| CA-125 | 13.18 | [26, 27, 32, 33, 39] |
| Abdominal ultrasonography | 65.17 | [22, 24–28, 30–35, 37–39] |
| Vaginal ultrasonography | 42.21 | [23, 31, 36–38] |
| Nursery (1st visit) | 26.49 | [22–39] |
| Nursery (subsequent visits) | 25.49 | [22–39] |
| Specialist physician (1st visit) | 144.41 | [22–39] |
| Specialist physician (subsequent visit) | 81.23 | [22–39] |
| Full blood count | 4.28 | [22, 24, 27, 32, 33, 38, 39] |
| Laparoscopy | 970.95 | [22–28, 34, 35] |
| Chest radiography | 19.95 | [22, 24–28, 30–39] |
| Abdominal MRI | 230.81 | [22–36, 38, 39] |
| Abdominopelvic CT scan | 126.19 | [22–30, 32, 36–39] |
| Hospitalizations and emergency department attendance | | |
| Ovarian cancer hospitalization | 5,960.23 | [87] |
| Emergency department attendance | 185.80 | [22–39] |
| Day hospital (price per minute) | 0,32 | [62] |
| Type of Surgery | | |
| Laparotomy | 1,793.61 | [22, 23, 27, 28, 32] |
| Abdominal total hysterectomy | 1,775.87 | [22, 23, 26, 28, 32, 35, 39] |
| Lymphadenectomy | 2,252.37 | [23, 28, 35] |
| Omentectomy | 2,698.49 | [28, 35] |
| Bilateral salpingoophorectomy | 1,775.87 | [22, 23, 27, 28, 35] |
| Palliative care services | | |
| Primary care (home care) | 60.93 | [22–25, 27–39] |
| Nursing care (home care) | 43.27 | [22, 24, 25, 27–37, 39] |
| Palliative care hospitalization | 5,291.41 | [88] |
| Palliative care services (home care) | 1,695.86 | [23] |
| Home care (price per hour) | 13.56 | [89] |

Table 2. Drug costs.

|  | List Price (LP) | LP - RDL +VAT |
| --- | --- | --- |
| CHEMOTHERAPY | | |
| PACLITAXEL | | |
| Paclitaxel EFG (6 MG/ML 1 vial concentrate for infusion 16.7 ML) | 130.89 € | 183.87 € |
| Paclitaxel EFG (6 MG/ML 1 vial concentrate for infusion 25 ML) | 196.34 € | 251.94 € |
| Paclitaxel EFG (6 MG/ML 1 vial concentrate for infusion 5 ML) | 39.27 € | 61.30 € |
| Paclitaxel EFG (6 MG/ML 1 vial concentrate for infusion 50 ML) | 392.67 € | 461.32 € |
| CARBOPLATIN | | |
| Carboplatin EFG (150 MG 1 vial 15 ML) | 22.92 € | 35.78 € |
| Carboplatin EFG (450 MG 1 vial 45 ML) | 68.76 € | 107.34 € |
| Carboplatin EFG (50 MG 1 vial 5 ML) | 7.64 € | 11.93 € |
| Carboplatin EFG (600 MG 1 vial 60 ML) | 91.68 € | 143.09 € |
| DOXORUBICINE PEGYLATED | | |
| Caelyx (20 MG 1 vial 10 ML) | 321.21 € | 387.00 € |
| BEVACIZUMAB | | |
| Avastin (25 MG/ML 1 vial 16 ML) | 1,272.89 € | 1,381.95 € |
| Avastin (25 MG/ML 1 vial 4 ML) | 341.71 € | 381.67 € |
| CISPLATIN | | |
| Cisplatin EFG (10 MG 1 vial 10 ML) | 2.23 € | 3.48 € |
| Cisplatin EFG (100 MG 1 vial 100 ML) | 22.30 € | 34.81 € |
| CISPLATIN EFG (50 MG 1 vial 50 ML) | 11.15 € | 17.41 € |
| GEMCITABINE | | |
| Gemcitabine EFG (1 G 1 vial concentrate for infusion 10 ML) | 43.70 € | 68.22 € |
| Gemcitabine EFG (1.5 G 1 vial concentrate for infusion 15 ML) | 65.55 € | 102.33 € |
| Gemcitabine EFG (2 G 1 vial concentrate for infusion 20 ML) | 87.40 € | 136.44 € |
| Gemcitabine EFG (200 MG 1 vial concentrate for infusion 2 ML) | 8.74 € | 13.64 € |
| TOPOTECAN | | |
| Topotecan EFG (1 MG/ML 5 vials concentrate for infusion 4 ML) | 407.65 € | 476.90 € |
| TRABECTEDIN | | |
| Yondelis (0.25 MG 1 vial powder) | 530.00 € | 609.35 € |
| Yondelis (1 MG 1 vial powder) | 1.994.00 € | 2,131.91 € |
| DOCETAXEL | | |
| Docetaxel EFG (20 MG/ML 1 vial concentrate for infusion 1 ML) | 43.97 € | 68.64 € |
| Docetaxel EFG (20 MG/ML 1 vial concentrate for infusion 4 ML) | 175.88 € | 230.66 € |
| Docetaxel EFG (20 MG/ML 1 vial concentrate for infusion 8 ML) | 351.76 € | 418.78 € |
| PALLIATIVE CARE | | |
| MIDAZOLAM | | |
| Midazolam Hospira (5 MG/ML 50 ampoules 3 ML) | 16.77 € | 26.18 € |
| MORPHINE | | |
| Mst Continus (100 MG 60 modified release tablets) | 47.14 € | 73.59 € |
| Morfina Braun (10 MG/ML 10 ampoules 1 ML) | 1.93 € | 3.01 € |
| DIAZEPAM | | |
| Diazepan Stada (10 MG 30 tablets) | 1.23 € | 1.92 € |
| Valium (10 MG 6 ampoules 2 ML) | 1.67 € | 2.22 € |
| SCOPOLAMINE | | |
| Escopolamina Braun (0.5 MG/ML 100 ampoules 1 ML) | 117.00 € | 169.43 € |
| HALOPERIDOL | | |
| Haloperidol Esteve (2 MG/ML oral drops 30 ML) | 1.60 € | 2.50 € |
| Haloperidol Esteve (5 MG 5 ampoules 1 ML) | 1.90 € | 2.52 € |
| LACTULOSE | | |
| Lactulosa Lainco EFG (666 MG/ML oral solution 10 bottles 800 ML) | 37.40 € | 58.38 € |
| SALINE SOLUTION | | |
| Suero fisiológico Vitulia (0.9% 10 solution 1000 ML) | 12.69 € | 16.84 € |

RDL: Royal decree law; VAT: value added tax; Reference: [58]*.*

Table 3. Doses of chemotherapy by disease stage and infusion time necessary for each drug

|  | Doses (mg) | | | | Infusion Time (hour) |
| --- | --- | --- | --- | --- | --- |
|  | Stage I | Stage II | Stage III | Stage IV |  |
| Paclitaxel | 292.35 | 297.49 | 292.35 | 295.60 | 3.5 |
| Carboplatin | 477.15 | 466.13 | 445.30 | 436.30 | 1.5 |
| Doxorubicin (monotherapy) | 66.82 | 68.00 | 66.82 | 67.56 | 2.0 |
| Doxorubicin (in combination) | 50.12 | 51.00 | 50.12 | 50.67 | 2.0 |
| Bevacizumab* | 975.00 | 1,005.00 | 975.00 | 990.00 | 2.0 |
| Gemcitabine | 1,670.58 | 1,699.95 | 1,670.58 | 1,689.12 | 1.0 |
| Cisplatin | 125.29 | 127.50 | 125.29 | 126.68 | 18.5 |
| Docetaxel | 125.29 | 127.50 | 125.29 | 126.68 | 1.5 |
| Topotecan | 6.68 | 6.80 | 6.68 | 6.76 | 1.0 |
| Trabectedin | 1.84 | 1.87 | 1.84 | 1.86 | 3.5 |

Note: (*) Dose of bevacizumab: 15mg/kg. References: [46–54].

Table 4. Market Shares.

|  | 1^st^ line | | 2^nd^ line | 3^rd^ line | 4^th^ line | 5^th^ line | 6^th^ line | 7^th^ line |
| --- | --- | --- | --- | --- | --- | --- | --- | --- |
|  | Stages I and II | Stages III and IV |  |  |  |  |  |  |
| Carboplatin | 100% | 79.9% | 75% | 18.3% | 26% | 25.5% | 24.4% | 23.2% |
| Paclitaxel | 92.3% | 48.7% | 49% | 27.3% | 39.6% | 42.1% | 43.8% | 45.1% |
| Doxorubicin (monotherapy) | - | 4.9% | 6% | 50% | 29.4% | 26.7% | 24.9% | 23.4% |
| Doxorubicin (in combination) | - | 12,8% | 10% | - | - | - | - | - |
| Bevacizumab | - | 36.8% | 27% | 9% | 13.6% | 16.6% | 19.4% | 21.9% |
| Gemcitabine | - | 23.2% | 18% | 20% | 26.9% | 25.8% | 24.5% | 23.2% |
| Cisplatin | - | 4.8% | 3% | - | - | - | - | - |
| Docetaxel | 7.70% | 1.6% | - | - | - | - | - | - |
| Topotecan | - | 1.6% | 2% | 2.67% | 4.2% | 5.4% | 6.8% | 8.3% |
| Trabectedin | - | 4% | - | - | - | - | - | - |

Resource: Own elaboration based on Drug Shares (Ipsos Monitor 2015) [45].

Table 5. Use of drugs in palliative care phase.

|  | Before terminal phase [69, 70] | | During terminal phase [71] | |
| --- | --- | --- | --- | --- |
|  | Use of drugs | Daily dose (mg/mL) | Use of drugs | Daily dose (mg/mL) |
| Midazolam | 5% | 46.80 | 100% | 179.4 |
| Morphine | 87% | 100 | 26% | 30 |
| Diazepam | 23% | 10 | 25% | 10 |
| Scopolamine | 36% | 0.9 | 45% | 0.9 |
| Haloperidol | 27% | 8 | 43% | 8 |
| Lactulose | 21% | 30 | - | - |
| Saline solution | - | - | 100% | 1,000 |

Table 6. Women employed and average annual salaries.

| Range of age | Women employed (%) [82] | Average annual salaries (€) [80] |
| --- | --- | --- |
| 20 - 24 | 30.30% | 10,603.52 € |
| 25 - 29 | 59.16% | 15,019.71 € |
| 30 - 34 | 67.64% | 18,435.40 € |
| 35 - 39 | 68.56% | 20,324.74 € |
| 40 - 44 | 64.72% | 20,953.82 € |
| 45 - 49 | 61.21% | 21,101.20 € |
| 50-54 | 58.10% | 21,786.77 |
| 55-59 | 48.14% | 22,482.92 |
| 60-64 | 28.99% | 20,460.53 |
